# Supplementary material for: Deletion of wfs1 Impairs Oligodendrocyte Precursor Cells Dorsal Distribution and Myelination Through the wfs1-hmgcs1 Axis in Zebrafish
Source: Biology (Basel). 2026 Aug 16;15(16):1402. doi: 10.3390/biology15161402 (PMC13510151; doi:10.3390/biology15161402)
Supplement: Supplementary file 1 [file biology-15-01402-s001.zip › File S1-Transcriptome sequencing data and differentially expressed genes (DEGs) in wfs1-- zebrafish at 5 dpf/2.KEGG/wfs1KOvsWT/DOWN/src/dre04217.html]

dre04217


- dre:564295

- Down regulated genes

564295\_2(-1.27307321576804)

- dre:403131

- Down regulated genes

403131(-4.42862381698358)

- dre:373107

- Down regulated genes

373107\_1(-7.07514976036473)

- dre:561021

- Down regulated genes

561021\_1(-4.68369619674599)

- dre:337413

- Down regulated genes

337413(-9.71650516648186)
- dre:368481

- Down regulated genes

368481(-2.13889418398421)

- dre:110438871

- Down regulated genes

321622(-1.53203125771205)

- dre:100149436

- Down regulated genes

100149436\_1(-1.86860481077668)

- dre:559768

- Down regulated genes

559768(-4.00475710388481)
- dre:558816

- Down regulated genes

558816(-1.68738667729536)
- dre:100126128

- Down regulated genes

100126128(-1.3085831331184)

- dre:100148818

- Down regulated genes

100148818(-1.62621488580639)

- dre:336426

- Down regulated genes

336426\_1(-6.2697721375186)
- dre:550542

- Down regulated genes

550542\_1(-6.05661837629532)

Close
